# Supplementary material for: Vesicular Transport Mediated by Endoplasmic Reticulum Stress Sensor BBF2H7 Orchestrates Melanin Production During Melanogenesis
Source: Int J Mol Sci. 2026 Jan 3;27(1):501. doi: 10.3390/ijms27010501 (PMC12786941; doi:10.3390/ijms27010501)
Supplement: Supplementary file 1 [file ijms-27-00501-s001.zip › ijms-4029173-Supplementary Legend-final.pdf]

Supplemental Figure S1. Analysis for expression levels of *Tyrosinase* using publicly available dataset GSE45226. Expression levels of *Tyrosinase* in melanocytes derived from human embryonic stem cells (normal) and in melanocytes derived from pluripotent stem cells of patients with oculocutaneous albinism (patient-1 and patient-2), obtained from the gene expression omnibus dataset GSE45226, shown as a box plot (n = 3).

Supplemental Figure S2. Pathway and gene ontology analyses using publicly available dataset GSE45226. Genes showing more than a two-fold change (increase or decrease) in expression between normal melanocytes and patient-1 or patient-2 melanocytes ($P < 0.05$) were used for pathway and gene ontology analyses. (**A**) Pathway analysis of differentially expressed genes in the comparison between normal and patient-2. (**B**) Gene ontology (biological process) analysis of the comparison between normal and patient-2. "Protein transport" is the eighth most enriched category (fold enrichment: 2.1). (**C**) Gene ontology (cellular component) analysis of the comparison between normal and patient-1. (**D**) Gene ontology (cellular component) analysis of the comparison between normal and patient-2. The fourth, tenth and twelfth largest categories are "secretory vesicle" (fold enrichment; 2.1), "vesicle" (fold enrichment; 1.7) and "intracellular vesicle" (fold enrichment; 1.7), respectively.

Supplemental Figure S3. BBF2H7 directly binds to CRE-binding site within the *Sec23a* promoter. (**A**) Schematic of the *Sec23a* promoter and the annealing sites of the primer set

used in the chromatin immunoprecipitation assay. Numbers indicate the distance from the transcriptional start site. CRE, cyclic AMP response element. (**B**) The results of PCR amplification of the *Sec23a* promoter containing the CRE-binding site after immunoprecipitation by anti-BBF2H7 antibody (n = 3). Mean ± SD. *P < 0.05.

Supplemental Figure S4. BBF2H7 pathway does not have any crosstalk with IRE1 or ATF6 pathways. (**A**) Western blot analysis of IRE1 and ATF6 in B16 cells transfected with non-targeting (nt) siRNA, or si*Bbf2h7*. (**B**) Western blot analysis of spliced form of X-box binding protein 1 (XBP1s) in B16 cells treated with α-MSH and transfected with si*Bbf2h7*. To inhibit IRE1 activity, cells were treated with 4μ8c. (**C**) Western blot analysis of ATF6 in B16 cells treated with α-MSH and transfected with si*Bbf2h7* and si*Atf6*. (**D**) Melanin content in B16 cells treated with α-MSH. Cells were treated with 4μ8c or transfected with si*Bbf2h7*, or si*Atf6* (n = 3). N.S., not significant. Mean ± SD.
