# Supplementary figures and images for: Vesicular Transport Mediated by Endoplasmic Reticulum Stress Sensor BBF2H7 Orchestrates Melanin Production During Melanogenesis
Source: Int J Mol Sci. 2026 Jan 3;27(1):501. doi: 10.3390/ijms27010501 (PMC12786941; doi:10.3390/ijms27010501)

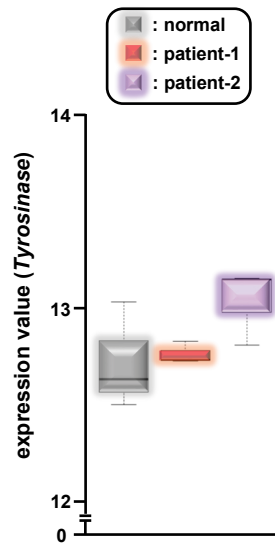

Supplemental Figure S1

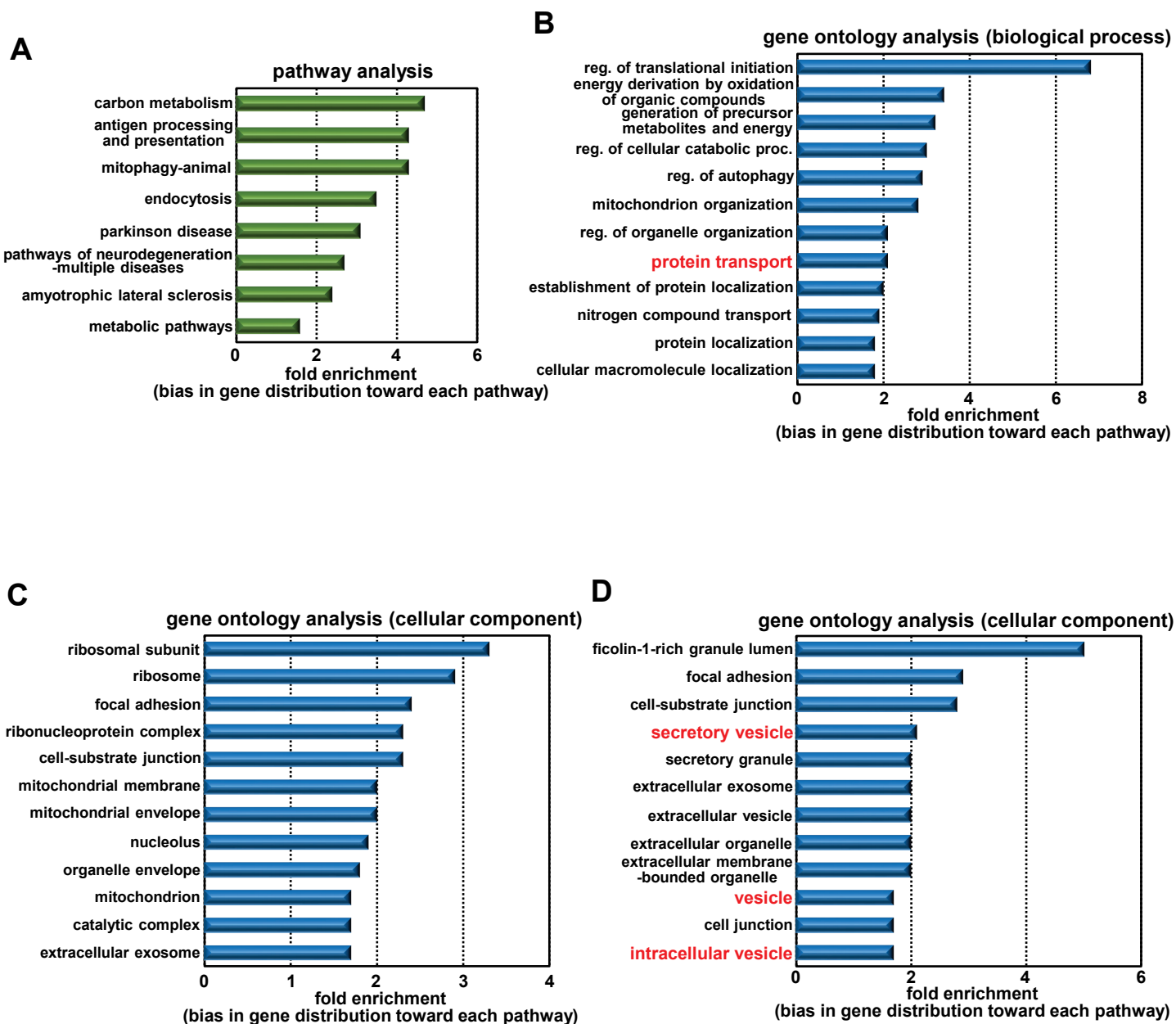

**A**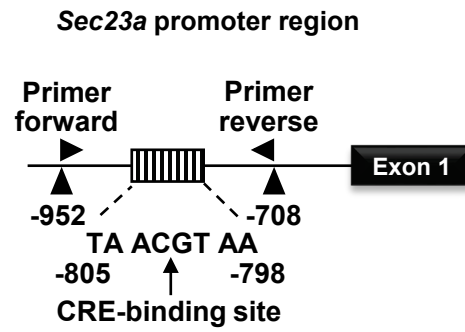**B**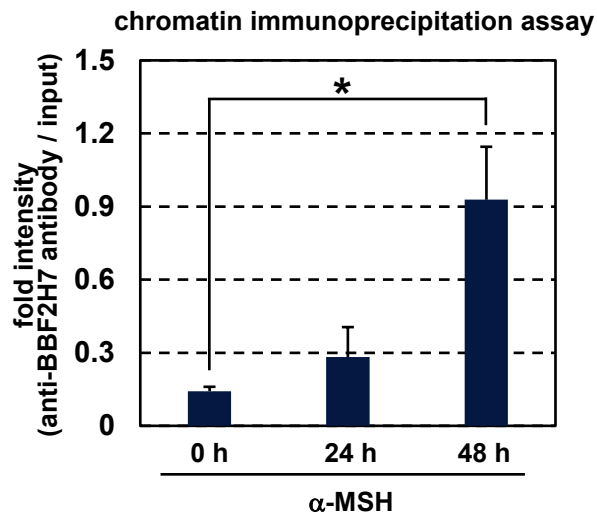

**A**

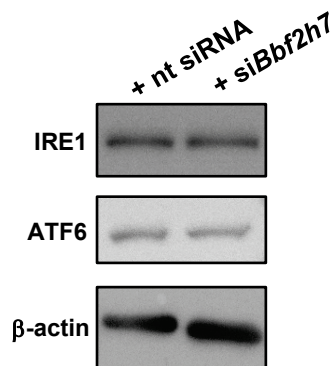

**B**

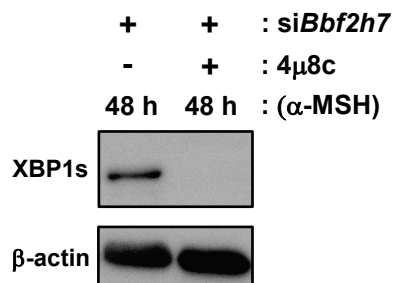

**C**

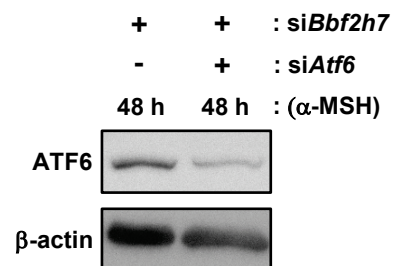

**D**

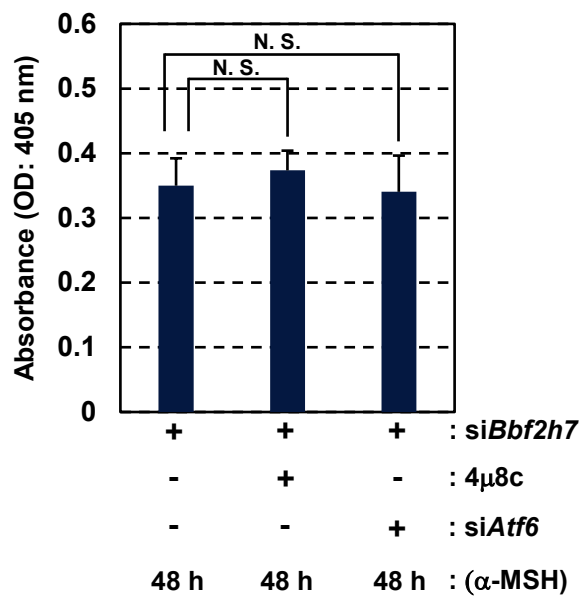

Supplement: Supplementary file 1 [file ijms-27-00501-s001.zip › ijms-4029173-Supplemental Figures-final.pdf]
